# On the Myths of Indicator Species: Issues and Further Consideration in the Use of Static Concepts for Ecological Applications

**DOI:** 10.1371/journal.pone.0078219

**Published:** 2013-10-16

**Authors:** Michael L. Zettler, C. Edward Proffitt, Alexander Darr, Steven Degraer, Lisa Devriese, Clare Greathead, Jonne Kotta, Paolo Magni, Georg Martin, Henning Reiss, Jeroen Speybroeck, Davide Tagliapietra, Gert Van Hoey, Tom Ysebaert

**Affiliations:** 1 Leibniz Institute for Baltic Sea Research, Warnemünde, Rostock, Germany; 2 Department of Biological Sciences, Florida Atlantic University, Ft. Pierce, Florida, United States of America; 3 Royal Belgian Institute of Natural Sciences, Marine Ecosystem Management Section, Brussels, Belgium; 4 Institute for Agricultural and Fisheries Research, Bio-Environmental Research, Oostende, Belgium; 5 Marine Scotland Science Marine Laboratory, Aberdeen, Scotland, United Kingdom; 6 Estonian Marine Institute, University of Tartu, Tallinn, Estonia; 7 Consiglio Nazionale delle Ricerche, Istituto per l’Ambiente Marino Costiero (CNR-IAMC), Oristano, Italy; 8 University of Nordland, Faculty of Biosciences, Aquaculture, Bodø, Norway; 9 Research Institute for Nature and Forest, Brussels, Belgium; 10 Consiglio Nazionale delle Ricerche, Istituto di Scienze Marine (CNR-ISMAR), Venezia, Italy; 11 Senckenberg am Meer, Marine Research Department, Wilhelmshaven, Germany; 12 Netherlands Institute of Sea Research (NIOZ), Yerseke, The Netherlands; 13 Wageningen University, Institute for Marine Resources and Ecosystem Studies (IMARES), Yerseke, The Netherlands; Universidade Federal do Rio de Janeiro, Brazil

## Abstract

The use of static indicator species, in which species are expected to have a similar sensitivity or tolerance to either natural or human-induced stressors, does not account for possible shifts in tolerance along natural environmental gradients and between biogeographic regions. Their indicative value may therefore be considered at least questionable. In this paper we demonstrate how species responses (i.e. abundance) to changes in sediment grain size and organic matter (OM) alter along a salinity gradient and conclude with a plea for prudency when interpreting static indicator-based quality indices. Six model species (three polychaetes, one amphipod and two bivalves) from the North Sea, Baltic Sea and the Mediterranean Sea region were selected. Our study demonstrated that there were no generic relationships between environment and biota and half of the studied species showed different responses in different seas. Consequently, the following points have to be carefully considered when applying static indicator-based quality indices: (1) species tolerances and preferences may change along environmental gradients and between different biogeographic regions, (2) as environment modifies species autecology, there is a need to adjust indicator species lists along major environmental gradients and (3) there is a risk of including sibling or cryptic species in calculating the index value of a species.

## Introduction

“Bioindicators” and “indicator species” are terms commonly used in ecological assessment and generally denote the use of observations on the status of organisms and communities to draw conclusions on environmental quality [1-7]. The knowledge of the environmental preferences of an organism or species can be applied in both temporal and spatial comparisons to detect changes in abiotic conditions or to assess the status of the habitat. The usefulness of species for the detection of both naturally and anthropogenically induced changes renders them an indicator status. Although applied in the assessment of marine systems for more than 30-40 years [8,9], bioindicators gained importance in two periods, during the 1960’s and even earlier [2-7] due to the emergent awareness of environmental pollution. Also the beginning of this century there was an increasing demand for the assessment of the ecological status of coastal and marine waters. This is exemplified by the EU Water Framework Directive (WFD; 2000/60/EC) and the Marine Strategy Framework Directive (MSFD; 2008/56/EC), in which the sensitivity/tolerance classification of species play an essential role [10]. 

Indicator species can signal a change in the biological condition of a particular ecosystem, and thus may be used to diagnose the health of an ecosystem. Ecological condition in aquatic environments is often evaluated by studying the sedimentary habitat and associated benthic communities. This means looking for species which, due to their autecological requirements, are constrained by narrow environmental conditions and are therefore typical and/or indicative for these conditions [3,11]. Such species seem to have a high indicator power. However, the concept of "indicator species" is slightly different from what we might call the "degree of sensitivity" of a species. In the first case a species is used because its presence, absence, or abundance indicates a precise suite of environmental conditions, for which it has tolerance or preference. Few species are used in this kind of assessment. The second case is derived from the “community composition” approach, where all the species potentially present in an environment are used to formulate the assessment. Species are listed and a score, used for the computation of a biotic index, is assigned to each species. The indicator value is then given by the proportion of the different species in the community composition [12-14]. It is essential however that the response of a particular indicator species to changes in an environmental variable is understood. This allows the level of the environmental pressure to be estimated. 

Benthic indicator species not only provide information on benthic environmental conditions, but also on the perturbations affecting a community when grouped into categories such as sentinel, tolerant or opportunistic species [3,12-15]. Species can consequently be classified into ecological groups defined by their sensitivity or tolerance to given disturbances or stresses, caused by e.g., organic enrichment, industrial waste, thermal pollution but also by natural factors (e.g., salinity gradients displayed in transitional water bodies). Thus, we expect that such generic bioindication-based assessment schemes are not likely to work or are highly biased when applied over broad spatial extents. This is also supported by specific examples. Brackish or diluted water bodies like the Baltic Sea were often labelled “bad” by certain indices due either to the absence of “good” indicator species or the indices were not adjusted for these conditions [16,17]. In these naturally stressed ecosystems, where natural variability occurs on different spatial (e.g. along gradients) and temporal (regular and coincidental) scales, it is hard to differentiate natural and anthropogenic stresses [18]. Similarly another example of biased classification has been raised in the Mediterranean lagoons that could be related to the inherently reduced number of species occurring in these highly variable and eutrophic systems [18-20].

The use of sensitivity/tolerance lists of benthic faunal species to define the ecological status of waters is generally accepted and globally applied [21-26] although the majority of these lists are built one on another and based on best professional judgement [20]. These lists are useful tools and have been updated in the last decade by the completion of the worldwide species list and an improved understanding of the autecology of many species. Reference species lists associated with static “indicator values” have been used more often than other methods [24]. Caution is required however, because each species is categorised into a single ecological category. Both from terrestrial and aquatic studies we know that many species can change their life history strategy or autecology requirements along environmental gradients [27-29]. It is generally accepted that sexes and age classes may differ in ecologically significant ways. However, phenotypic variation among individuals can generate variation in ecological attributes even within a sex and age class, including, for example abiotic tolerances, resource use, or competitive ability (see [29] for references). 

If aspects of the preferences and/or tolerances of a species change over typically encountered environmental gradients, this can affect the species’ sensitivity, and therefore, its indicative value for other factors such as pollution [17,30]. Further, the ability of a species to serve as an indicator may depend on its position along an environmental gradient. Moreover species may be intrinsically different in their ability to discriminate particular environmental conditions. 

These problems have been faced since the initiation of benthic bioindication during the first half of the last century [15,31-35]. 

Following this reasoning and examples in this paper, the use of fixed reference lists needs to be reconsidered, especially in areas with strong salinity gradients, like estuaries or the Baltic Sea [16], or eutrophic systems like Mediterranean lagoons [19]. The interactions between biotic and environmental factors and the adaptive behaviour of species have to be considered in a species sensitivity/tolerance reference list, where the diagnostic power of a species would be linked to a specific ecological zone/environment and to its discriminating power. In this paper we tested the hypothesis that alterations in species’ environmental preferences and tolerances change along environmental gradients, using a large macrobenthic dataset spanning different biogeographical regions and different environmental conditions. We hypothesise that (1) relationships between organic enrichment and sediment grain size and biota change along major environmental gradients and (2) such relationships also vary among the European seas. This study is a step forward in disentangling co-varying effects, using salinity as the response variable for selected ‘test-species’, and organic matter content (OM) and grain size composition as a proxy of substrate and habitat characteristics (e.g. organic enrichment, sheltered vs. exposed waters). Based on the results, some recommendations are given to apply when using indicator species lists for ecological quality assessments.

## Materials and Methods

### Research strategy

The tolerance/sensitivity values of species have been developed based on life history features using a variety of techniques. Several studies have applied different methods to classify the sensitivity or tolerance of benthic organisms to various degrees of disturbance. These were usually based on best professional judgement (including literature review and experimental research) [24,36]. Another approach was the quantitative (or mathematical) determination of the tolerance values of benthic species to environmental disturbance [17,26,37]. These values were commonly combined or used within a biotic index to allow the assessment of gradients of environmental conditions [38,39]. However, whether these gradients were of anthropogenic or natural origin was generally not considered in these investigations. Salinity gradients predominantly control the distribution of organisms on the coast, in estuaries and lagoons as well as in the Baltic Sea [40,41]. Salinity is a key environmental factor, which defines structural and functional characteristics of aquatic biota [42]. Moreover, salinity is a common confounding factor in nearly all coastal environments in which human impacts are being assessed [43]. It is acknowledge that species' tolerances/preferences change along the multitude of natural and human-induced environmental variables, the quantification of the species tolerances/preferences along strong environmental gradients will allow the use of static lists to be put in context. In this study, salinity gradients were selected as the primary environmental gradient against which the preferences and tolerances of a set of species were quantified. The secondary variables, grain size distribution and organic matter content were selected as the main substrate characteristics that would influence the distribution of macrozoobenthic species [19,44]. 

### Data availability

This paper presents collaborative research based on a collation of existing data. Table 1 provides further details on the data sources (i.e. contact person, availability). Data are compiled into appendices (Appendix S1-S4).

**Table 1:** Number of samples, sampling period and availability of data used in the present study.

	**Great Britain**	**North Sea 1**	**North Sea 2**	**Wester-schelde**	**Zee-schelde**	**Belgian Beaches**	**Belgium Offshore**	**German Baltic**	**Estonia**	**Cabras Lagoon**	**Venice Lagoon**
**Number of samples**	**126**	**30**	**84**	**2089**	**104**	**343**	**131**	**1012**	**1549**	**76**	**285**
**Sampling period**	**2001-2011**	**2000-2004**	**2008-2011**	**2000-2008**	**1990-2011**	**1994-1995**	**2005-2010**	**2001-2011**	**2003-2009**	**2001-2004**	**1991-2002**
**Availability**	**On request**	**On request**	**On request**	**On request**	**On request**	**On request**	**On request**	**On request**	**On request**	**On request**	**On request**
**Responsible author**	**CG**	**HR**	**MLZ**	**TY**	**JS**	**SD**	**GVH**	**MLZ**	**JK**	**PM**	**DT**

**Figure 1:**
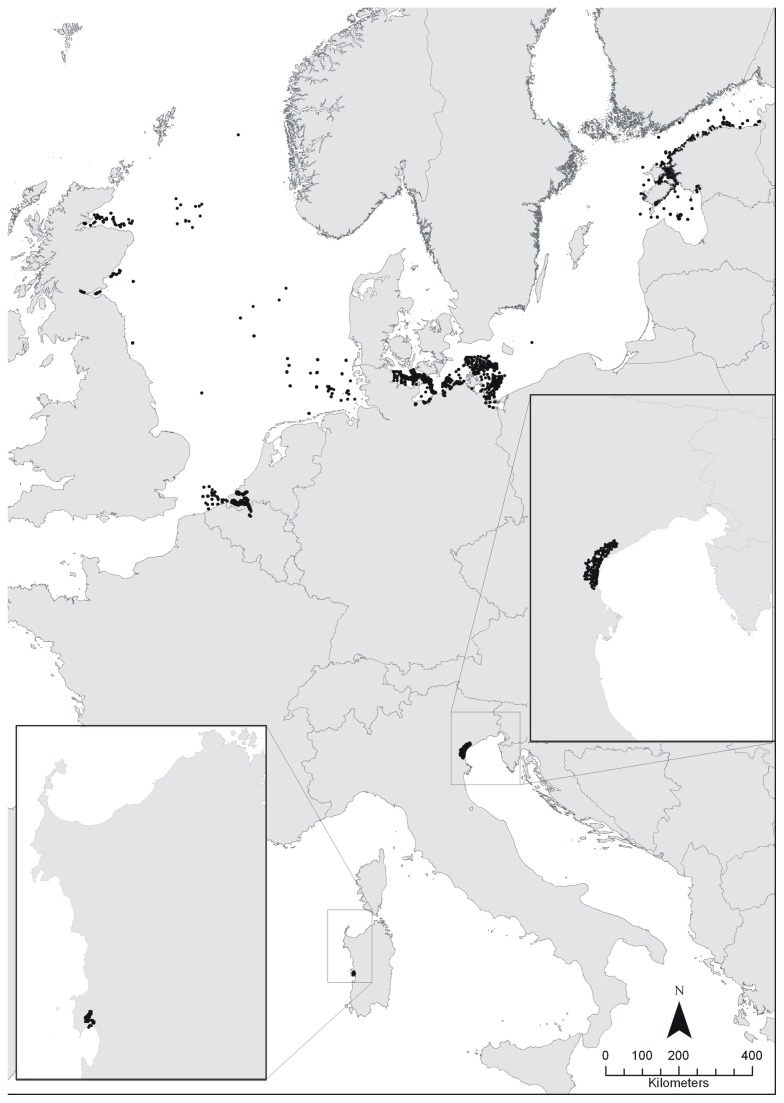
Map of sample locations included in this study.

Only sample data that contained information on salinity, substrate (sediment grain size, organic matter content) and the abundance of selected species (including zero-values indicating the absence of the species at an individual station) were retained for analysis. Overall, 5468 records from the North Sea and Baltic Sea and 361 records from the Mediterranean were available for this study (Tables 2 and 3). The data originate from nine case-studies and monitoring programs from around the North Sea and Baltic Sea, covering coastal and offshore waters of the UK, Belgium, the Netherlands, Germany and Estonia (Figure 1). In the Mediterranean Sea, data were available from two Italian lagoons, namely the Venice Lagoon and the Cabras Lagoon (Isle of Sardinia). These datasets, covering a wide range in salinity, were combined into two datasets (two geographical regions), i.e. a North Sea – Baltic Sea and a Mediterranean Sea database. 

**Table 2:** Data availability (loss on ignition (LOI) and median grain size) in the North and Baltic Sea.

**North & Baltic Sea**		**Great Britain**	**North Sea 1**	**North Sea 2**	**Westerschelde**	**Zeeschelde**	**Belgian Beaches**	**Belgium Offshore**	**German Baltic**	**Estonia**	**Overall**
**LOI**	**oligo**								72	55	**127**
	**β-meso**								362	1467	**1829**
	**α-meso**								209		**209**
	**β -poly**								278		**278**
	**α -poly**	4		3			11	9	75		**102**
	**euhalin**	113	27	81			332	91			**644**
**grain size**	**oligo**					11			72	46	**129**
	**β-meso**				88	93			269	1247	**1697**
	**α-meso**				523				184		**707**
	**β -poly**				591				225		**816**
	**α -poly**	4		3	739		11	11	75		**843**
	**euhalin**	60	30	81	87		332	120			**710**
**overall**	**oligo**					11			72	55	**138**
	**β-meso**				88	93			362	1494	**2037**
	**α-meso**				523				225		**748**
	**β -poly**				591				278		**869**
	**α -poly**	4		3	739		11	11	75		**843**
	**euhalin**	122	30	81	87		332	120			**772**

**Table 3:** Data availability (loss on ignition (LOI) and median grain size) in the Mediterranean Sea.

**Mediterranean**		**Cabras**	**Venice**	**Overall**
**LOI**	**meso**	38	22	**60**
	**poly**	24	103	**127**
	**eu**	14	160	**174**
**grain size**	**meso**	38	22	**60**
	**poly**	24	103	**127**
	**eu**	14	160	**174**
**overall**	**meso**	38	22	**60**
	**poly**	24	103	**127**
	**eu**	14	160	**174**

The salinity at each sampling location was either measured directly or taken from modelled data (Schelde estuary). At intertidal locations, salinity was measured from the receding water immediately after the sampling location became exposed. In this study, a reduced six-level Venice salinity system was used: euhaline zone >34 to 30, α-polyhaline zone 25 to 30, β-polyhaline zone 18 to 25, α-mesohaline zone 18 to 10, β-mesohaline zone 10 to 5 and oligohaline zone 5 to 0.5 (see [45] for details). However, due to the comparably small data-set, Mediterranean data were subdivided only into three salinity classes (poly-, meso- and oligohaline, Table 3). 

Grain size distribution was taken into account as median grain size (d50 in µm) (Tables 2 and 3). Within the North/Baltic Sea dataset, stations with d50 > 2000 µm were excluded, as these were scarce and available only for a single salinity class. Overall, 4951 records were used. Median grain size was available for all Mediterranean stations. Maximum median grain size was 964 µm. 

For most stations, sediment organic matter content (OM) was measured as loss on ignition (LOI in % of dry weight). For some stations in Belgium offshore waters (n=61) and the UK (n=126), LOI was calculated from the organic matter content (for Belgium the “dichromate method” for TOC [46]), LOI = 2.9324*organic matter content + 0.2979 (R^2^ = 0.85, p<0.01). The relationship was calculated from parallel measurements, available for Belgian waters (n = 39). Overall, 3559 data points with information on organic content of the sediment were included in the dataset. Previously proposed OM ranges were used as an indication of the level (low, moderate, high) of OM enrichment [19,44,47]. This is because OM in sediments can be a stressor when it is in excess, while at low to moderate levels (ca. up to 8-10%) it is an important food source for benthic animals [44,47]. 

### Species selection

Species selected had to comply with the following criteria: a) wide distribution range (occurring in all study areas), b) present in at least three salinity classes and c) the species should be used in current indices and (at least partially) be classified differently within the relevant indicators/metrics. Examples of such metrics include MarBIT, M-AMBI, ES(50)_0.05_-values in BQI and MarLIN, all commonly used in the study areas [10,16,18,24-26,30,37,39,48]. Consequently, six model/test species were selected: 3 polychaetes (*Ampharete baltica* Eliason, 1955, *Hediste diversicolor* (O.F. Müller, 1776), and *Travisia forbesii* Johnston, 1840), 1 crustacean (*Bathyporeia pilosa* Lindström, 1855) and 2 molluscs (*Cerastoderma glaucum* (Bruguière, 1789) and *Corbula gibba* (Olivi, 1792)) (Table 4).

**Table 4:** Sensitivity classification of selected species in European assessment systems and sensitivity against nutrient load (MarLIN-database) referring to the following references [17,24,36,48].

**species**	**MARBIT (German WFD-tool)**	**AMBI/M-AMBI**	**BQI (adaptation southern Baltic) - range E(S50)0.05**	**MarLIN**
***Ampharete baltica***	indifferent (N)	indifferent (II)	tolerant to sensitive (6.6-10.5)	not included
***Bathyporeia pilosa***	very sensitive (O)	very sensitive (I)	tolerant (4.5-6.9)	not included
***Cerastoderma glaucum***	very sensitive (O)	tolerant (III)	tolerant (5.7-6.6)	nutrient load: intermediate intolerance, low recoverability, high sensitivity
***Corbula gibba***	indifferent (N)	very tolerant (IV)	tolerant to sensitive (5.9-9.4)	*C. gibba* are indicative of unstable environments such as ones with low oxygen levels and areas of eutrophication within the Mediterranean [87]
***Hediste diversicolor***	tolerant (T)	very tolerant (IV)	tolerant (5.2-6.9)	high tolerance against changes in nutrient level, may benefit
***Travisia forbesii***	very sensitive (O)	very sensitive (I)	tolerant (6.3-6.9)	not included

The ecological requirements of these species are outlined below. The presence of muddy sediments seemed to be obligatory for the polychaete *A. baltica*, as small grain sizes are used for tube construction. No other sediment preferences or tolerances are known [49]. The polychaete *T. forbesii* is considered to be sediment-specific (regarding grain size and organic content) and therefore sensitive to changes in sediment composition [50]; sediments with especially high organic load are avoided. In contrast, *H. diversicolor* is known to be euryoecious, tolerating a wide range of salinity, temperature, and water quality [51]. *Bathyporeia pilosa* avoids very coarse sediments and prefers fine, somewhat muddy sand with at least 70% of the substrate finer than 210 µm [52]. It is more tolerant of fine particles in the sediment than other species of the same genus and is found in high densities on sediments with up to 25% silt. However, *B. pilosa* is often classified as “sensitive” to organic load and changes in sediment characteristics (see [52] for references). The bivalve *C. gibba* is widely distributed in European seas, from the Norwegian Sea southward to the Mediterranean and Black Seas (see [53] for references). *Corbula gibba* populations are distributed from low intertidal zones down to considerable depths. *Corbula gibba*, an infaunal species with a sedentary mode of life, inhabits soft bottom sediments very often mixed with molluscan shell fragments or thick muddy sand with admixed gravel and small stones [54]. Due to its high tolerance to a wide range of abiotic environmental disturbances, *C. gibba* is considered an indicator of environmental instability caused by pollution, low oxygen content, and/or increased turbidity [53]. The lagoon cockle *C. glaucum* is a euryhaline bivalve with a wide distribution along European coasts, ranging from the northern Baltic Sea to the Black Sea, the Caspian Sea and even Lake Aral, with salinities from 4 to 100 PSU [55]. In the Atlantic and the Mediterranean, *C. glaucum* typically inhabits closed brackish lagoons and estuaries.

### Statistics

The response of a species to an environmental gradient is affected by the simultaneous response of the species to multiple other parameters in natural systems. This increases heterogeneity and often masks the relationship between the gradient parameter and the response variable in ecological models focussing on single parameter gradients. Due to the interlinkage of environmental variables one possibility for dealing with this problem is to look at the maximum [56] or 95^th^ percentile [45] as the response variable. The modelling of an “outer envelope” [57] might be more appropriate than modelling the mean response as the outer envelope is less susceptible to the influence of other parameters. The advantage of the 95^th^ percentile over the maximum value is the lower sensitivity to outliers.

Quantile regression spline (QRS) models were used to reconstruct the response of the species along the gradient of the substrate parameter for each salinity class. The chosen procedure mainly follows the description in Anderson (2008) [57]. The QRS for the 95^th^ percentile were modelled for a set of four models per species and salinity class with a polynomial function of degree = 2, 3, 4 and 5. The quantile regression was modelled using the function ‘rq’ of the package quantreg [58] and a smoother was added using the function ‘bs’ [59] of the R statistical analysis software (R Development Core Team, 2012 [60]). Akaike’s information criterion (correction for small sample sizes AICc, [61]) was used for model selection. This automatic choice was modified if another model had a similar AICc value (within 2 units) and visually showed a better fit in the scatterplot of the data. Vertical dashed lines in the figures indicating the highest predicted density of the species in selected plots were interpreted as an estimated optimum of the species. QRS were modelled for all selected species. 

## Results

Neither consistent relationships in species responses to changes in sediment variables across salinity gradients, nor similar responses in different seas were found. The quantile regression spline models (quantile regression spline) did not show good fit with the data in three cases (*A. baltica*, *T. forbesii* and *C. glaucum*). In these three cases, simple bubble plots were used to illustrate non-significant trends. 

### Test species

**Figure 2:**
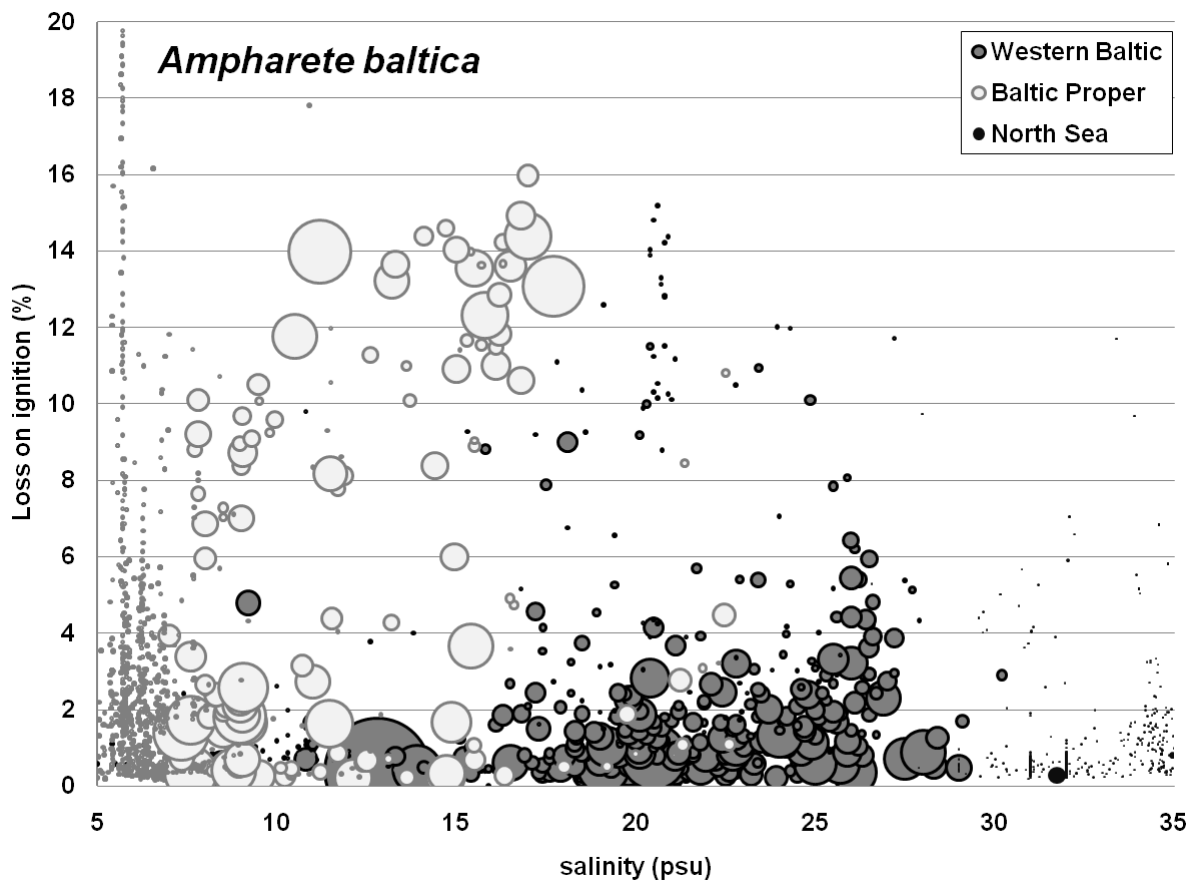
Abundance of *Ampharete baltica* in relation to bottom salinity and organic sediment content in three sea areas. The relative bubble size indicates the density of the species with smallest dots referring to species absence and largest bubble size to ca. 1700 ind/m^2^. Note that the symbol shading used in Figures 2, 3 and 5 is not consistent as different issues are shown.

*Ampharete baltica* was rare under fully marine conditions, but was found mainly in fine sandy bottoms with low to medium organic matter content in the Western Baltic (mainly polyhaline waters, Figure 2). However, in the Baltic Proper, sediment specificity seemed to become weaker as both low and high organic matter content sediments were inhabited, suggesting that the two different populations have adapted to specific conditions. It might be that the species exhibits high plasticity in its response to this environmental gradient.

**Figure 3:**
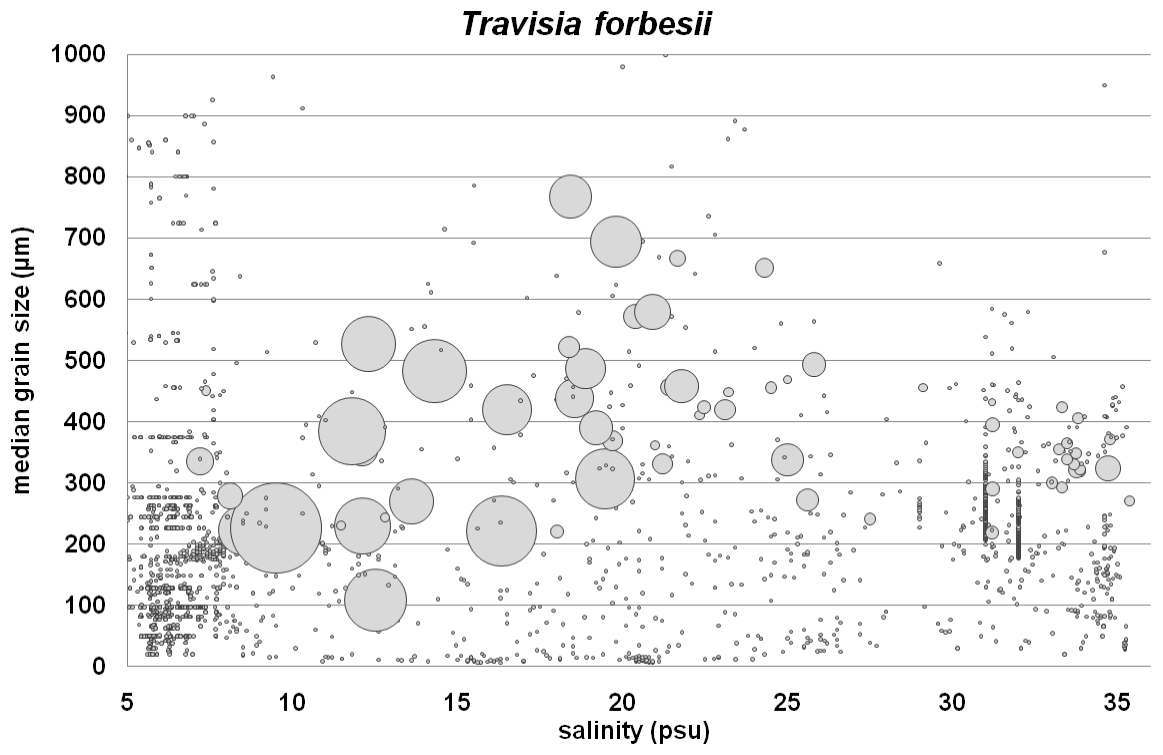
Abundance of *Travisia forbesii* along the full salinity gradient combining the North and Baltic Sea data set and along median grain size. Relative bubble size: species density (absent – ca. 670 ind/m^2^). Note that the symbol shading used in Figures 2, 3 and 5 is not consistent as different issues are shown.

*Travisia forbesii* is restricted to a narrow range of clean medium sands (d50: 250 - 500 µm) under fully marine conditions (Figure 3). In mesohaline waters, the species was found also in clean fine sands with a lower limit of about 100 µm. No occurrence was reported from coarse sediments in mesohaline waters (which may be attributed to data deficiency). 

**Figure 4:**
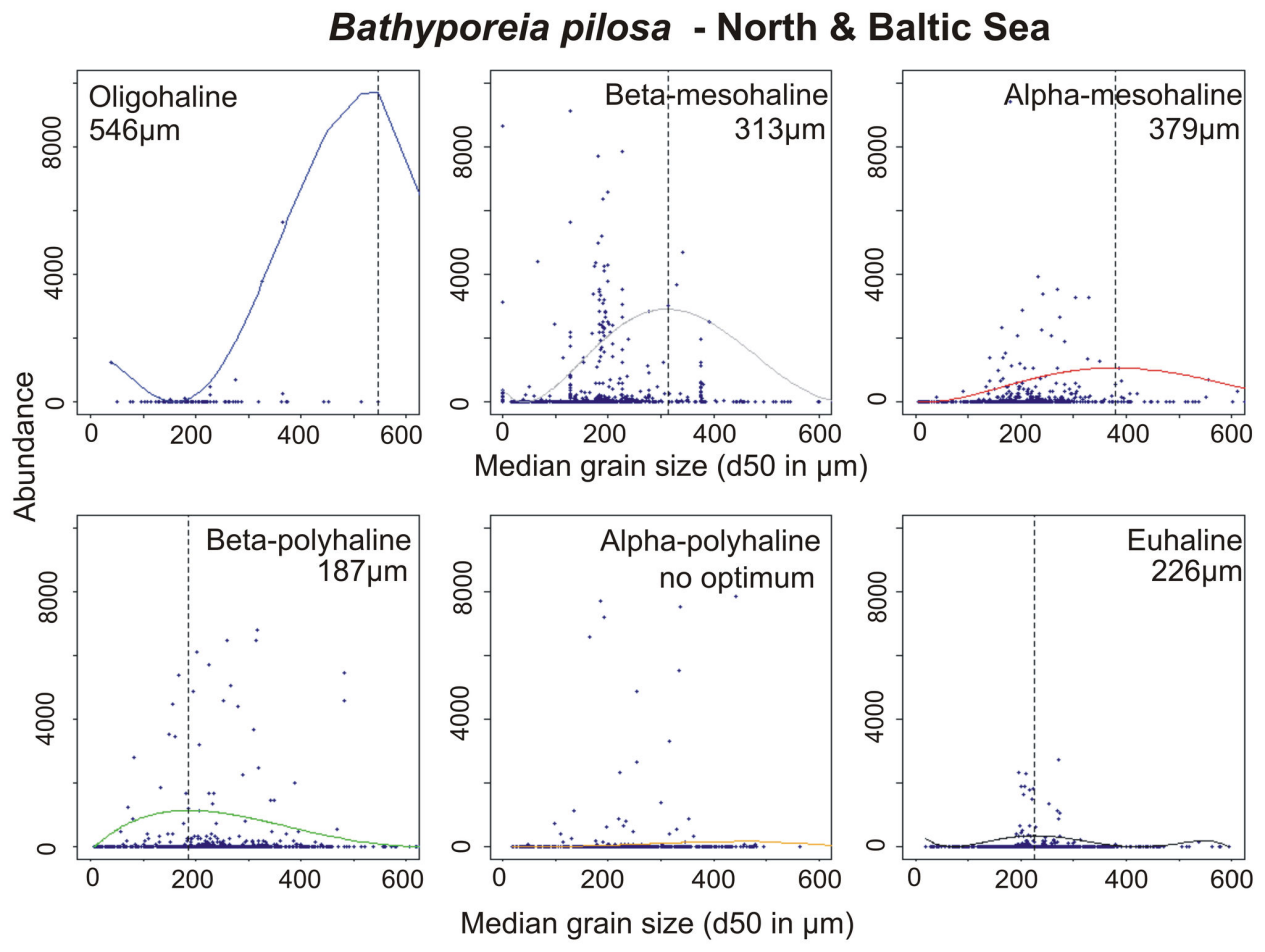
Abundance-response curves (95^th^ percentile QRS) of *Bathyporeia pilosa* along a sediment grain size gradient in six different salinity classes in the North Sea and the Baltic Sea. The dashed line marks the modelled optimum. Note that the y-axis had to be cut in some cases due to outliers.

*Bathyporeia pilosa* was present along almost the full spectra of available substrates from very fine sand to coarse sediments. Highest abundances were found on medium sand in ß-mesohaline areas and on fine sand (d50: 100-250 µm) in β-polyhaline and euhaline areas (Figure 4). In the remaining parts of the salinity gradient, medium sands seemed to be preferred. No optimum was found for the α-polyhaline section as high abundances occurred throughout the full substrate spectrum. 

**Figure 5:**
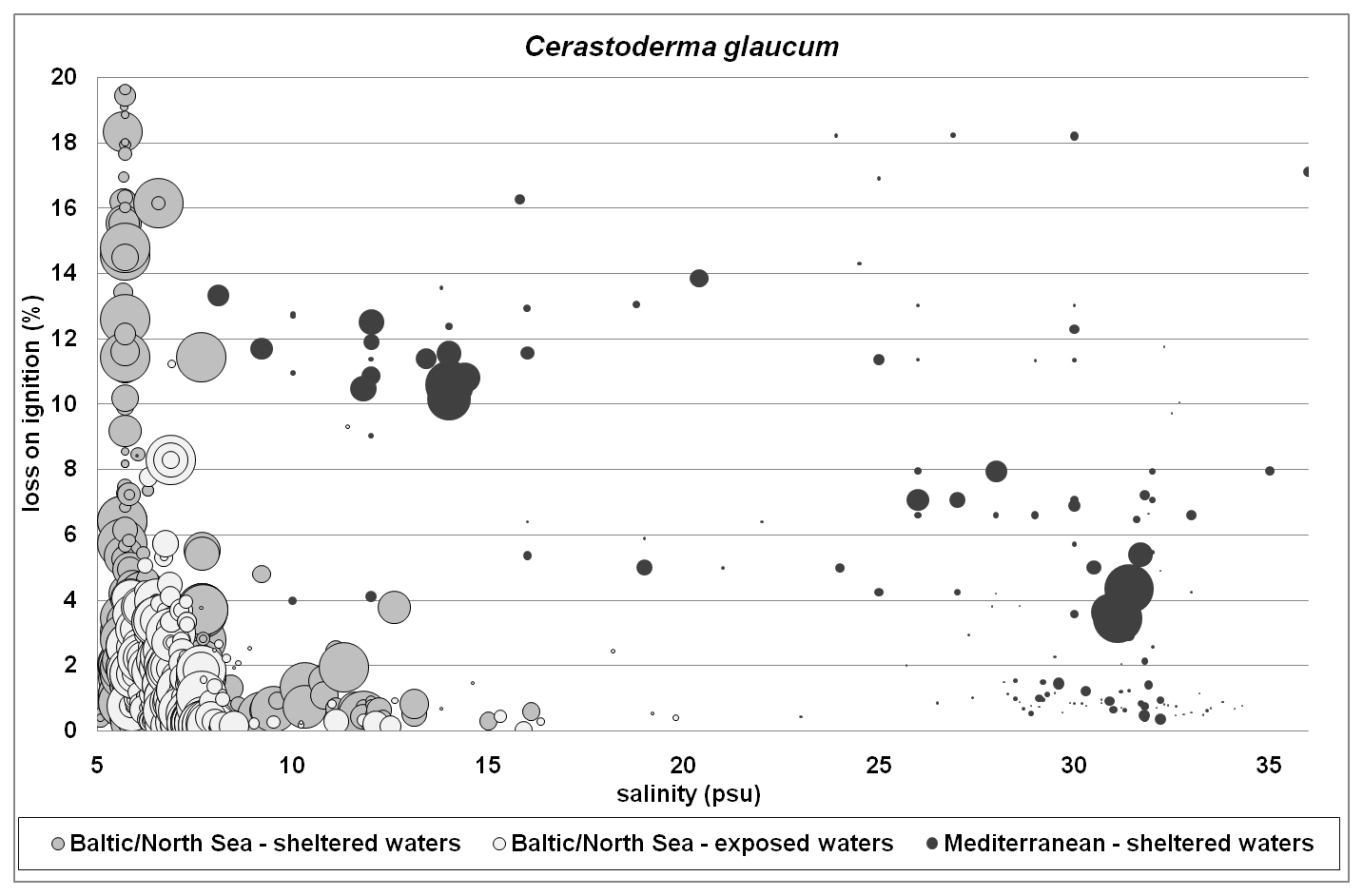
Abundance of *Cerastoderma glaucum* with relation to salinity and LOI both in the North/Baltic Sea and in the Mediterranean. Relative bubble size: density (absent - ca. 10000 ind/m^2^). Note that the symbol shading used in Figures 2, 3 and 5 is not consistent as different issues are shown.

The bivalve *C. glaucum* was restricted to β-mesohaline and lower α-mesohaline waters in the Baltic Sea. It showed obvious differences in the ability to deal with organic enrichment between sheltered (e.g., estuaries, lagoons and inner bights) and more exposed (e.g., outer bights and offshore) waters. In exposed areas, it rarely occurred on sandy substrates with a LOI > 5% (Figure 5). In sheltered areas, it seemed to be tolerant to organic enrichment, indicated by consistently high densities throughout the range covered (0-20%). Also in the Mediterranean lagoons, it was found throughout the full organic gradient, but with highest densities in sediments with an intermediate to high organic load (LOI 3-14%). Additionally, the Mediterranean *C. glaucum* was present throughout the full salinity range. The tolerance of *C. glaucum* to OM content is apparently dependent on the OM content to which different populations are normally exposed.

**Figure 6:**
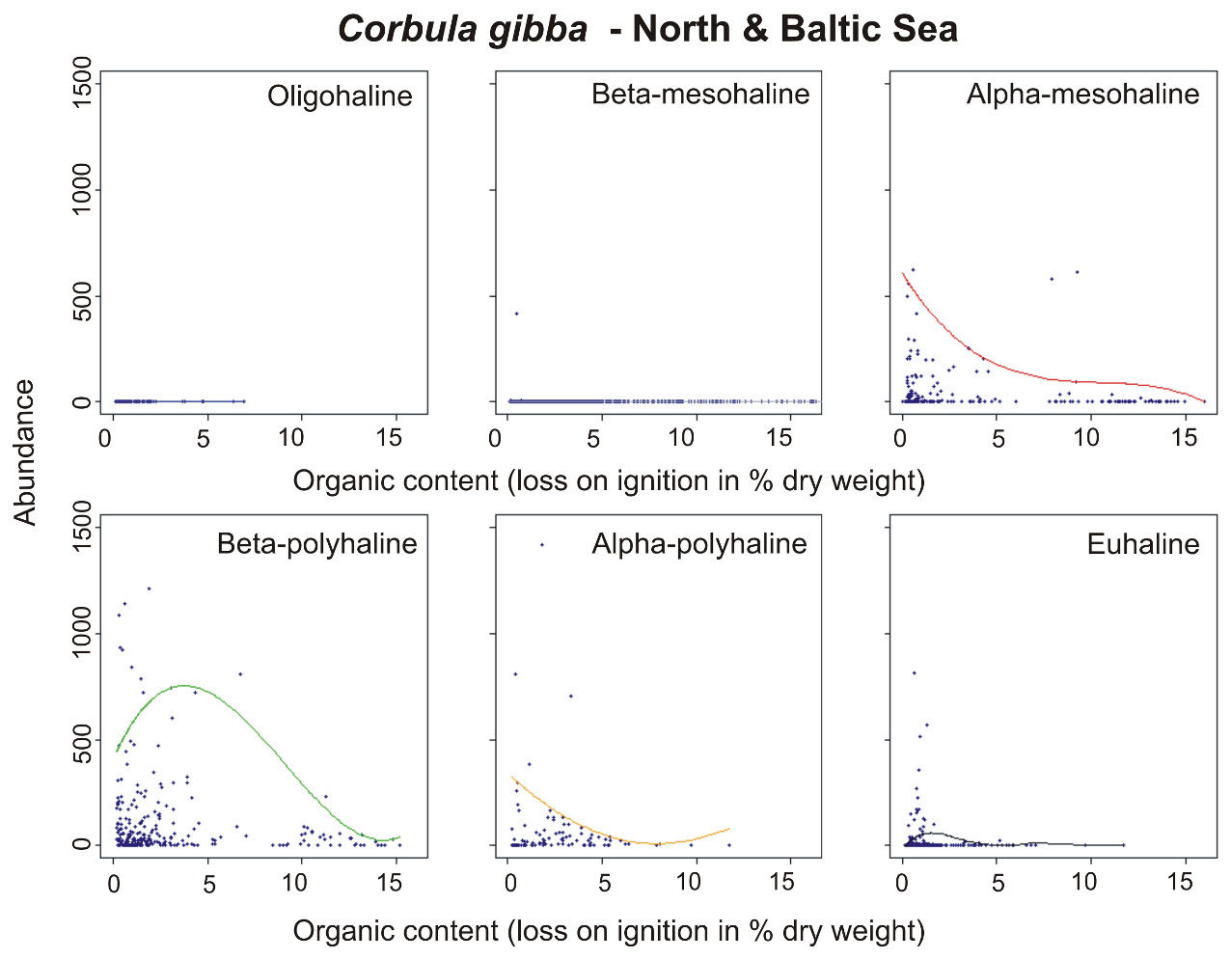
Abundance-response curves (95^th^ percentile QRS) of *Corbula gibba* along gradient of organic content (loss on ignition) in six different salinity classes in the North Sea and the Baltic Sea. The dashed line marks the modelled optimum. Note that the y-axis had to be cut in some cases due to outliers.

*Corbula gibba* was found from α-mesohaline to euhaline waters with abundances up to 1500 ind/m². This bivalve showed no clear habitat preferences, but high organic load was avoided (Figure 6). The species was not found on pure mud and seemed to prefer organic contents between 0 and 5 %. 

**Figure 7:**
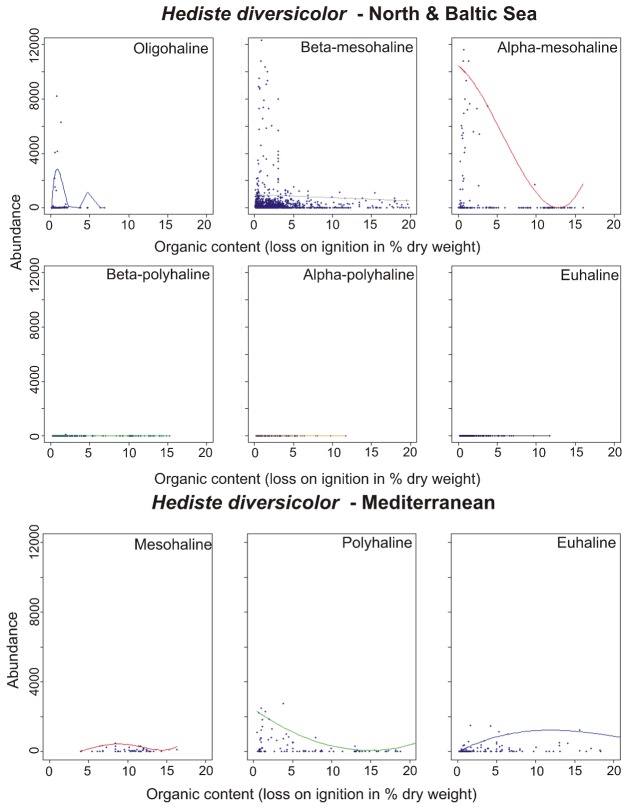
Abundance-response curves (95^th^ percentile QRS) of *Hediste diversicolor* along gradient of organic content (loss on ignition) in six different salinity classes in the North Sea and the Baltic Sea (top) and the Mediterranean (bottom). The dashed line marks the modelled optimum. Note that the y-axis had to be cut in some cases due to outliers.

The polychaete *H. diversicolor* is regarded as one of the most tolerant benthic species, settling under a large variety of conditions. Nevertheless, it reached its highest abundance in Baltic mesohaline waters in substrates with low organic load (Figure 7). Similar to *C. glaucum*, it populated different salinity classes in the Baltic and Mediterranean Sea, respectively. In the Baltic Sea, *H. diversicolor* occurred from oligohaline to ß-mesohaline, and in the Mediterranean Sea it preferred mesohaline to euhaline waters.

## Discussion

The distribution patterns of species are controlled by different factors of which the relative importance varies as a function of spatial scale [62]. The relative contribution of biotic interactions is considered to be more important for the community assembly processes of macrozoobenthos at smaller spatial scales, whereas life history traits and abiotic environmental conditions become relatively more significant at larger spatial scales. Nevertheless, the investigation areas covered within this study are characterised by large spatial and temporal variability in a number of abiotic environmental variables such as salinity, temperature, oxygen, ice disturbance, etc. Consequently, even at small spatial scales the formation of communities is largely determined by changes in these abiotic variables compared to biotic interactions or community assembly processes [18,63]. In this study only sediment characteristics and salinity were used to detect shifts in the tolerances or preferences of the selected species, while other variables with potential confounding effects were not included. For example, the distribution of trace metals is largely controlled by the granulometry and organic matter content of the sediments [64,65]. Thus, potential contamination from anthropogenic sources rather than sediment characteristics might be causally linked to our observations, but considering the large spatial scale of our study this appears less likely. However, the relationships within communities with abiotic environmental gradients demonstrate that species distributions are driven by influences that are multivariate in origin. This may lead to confusing interpretations on cause-effect relationships when based on information from too narrow sections along environmental gradients. Grémare et al. [30] therefore suggest that the use of a single sensitivity/tolerance list in different geographical areas (such as in AMBI) should be interpreted with care. The use of biological indicators as objective or subjective alternatives for assessing soft-bottom communities is discussed in Dauvin et al. 2012 [39]. Discrepancies between the values of indices are due to the dominance of species characterized as sensitive in one location and tolerant in another. Therefore, natural gradients in salinity (and others such as depth or sediment characteristics) may obscure the detection of macrobenthic responses to human pollution [66]. In systems with a large salinity gradient, the relationships between environmental factors, species distributions and biological traits (specific responses to certain salinities or sediments) may change considerably, as demonstrated in this study. Many species inhabiting variable environments are opportunistic, euryplastic, or transient. If species occur in a large range of environmental parameters (e.g., salinity) the whole range has to be taken into account when interpreting their value as ecological condition indicators. Detailed biogeographic, ecological, and genomic data would be required to accurately interpret indicator species responses. Since studies and data in this respect are rare, we have tried to highlight some issues relevant to the use of static indicators using existing data from monitoring programmes. Despite being aware of the limits of our approach, we believe that the information obtained is robust enough to draw reliable conclusions and inform other ecological applications.

This study has clearly supported the hypothesis that there was no generic response of species abundances to OM content or grain size (environmental “stressors”), along a salinity gradient. The abundance data for *A. baltica*, *C. glaucum*, *H. diversicolor* and *C. gibba* showed that tolerance to OM content changed with different salinity regimes. In other species however, such as *B. pilosa* and *T. forbesii*, it was the relative abundance in different sediment types that changed with salinity regime. These apparent differential preferences could be due to several factors. 

In the example of *A. baltica*, broader substrate preferences might be a consequence of lower interspecific competition in specific salinity regimes. Yet, also the existence of a similar and closely related, cryptic species with different sediment preferences in the Baltic Proper is possible. *Ampharete baltica* was considered by Jirkov (2001) [67] as a likely member of the *Ampharete lindstroemi* species complex. Maybe the validity of the species has to be checked [68]. Locally adapted genotypes can present an equally valid explanation for *A. baltica*, which would require molecular investigation. Alternatively, as a boreal or arctic species, temperature dependency might be stronger than its dependence on a certain type of sediment. 

The apparent sediment specificity of *T. forbesii* in full marine waters might be the result of interspecific competition. Otherwise, its ability to populate fine sands in mesohaline waters could be the result of local adaptation. Cryptic speciation might be at work here as well. 

The widespread occurrence of *H. diversicolor* in a wide variety of habitats might in fact also represent the distribution of several cryptic species in one reported taxon [69]. Potentially, one or several of those species might be regarded as being sensitive (e.g., highest abundances at low organic load). Several studies indicate that *H. diversicolor*, as considered in the present paper, is probably a mix of several cryptic and morphologically very similar species [70]. Consequently, *H. diversicolor* is most likely just as strongly differentiated into populations in the western Mediterranean as in the North Sea and Baltic Sea. However, these molecular studies have not yet been translated into an adapted taxonomy for *H. diversicolor*. As *H. diversicolor* is just one example, it is likely that the same problem exist for several other species, currently identified as sensitive or tolerant according to different sensitivity lists (e.g. *C. glaucum* – [71]). The autecology of sibling and cryptic species can differ completely, as they may occupy different niches [72-74]. Although there is considerable variability in the distribution of cryptic species within and between waterbodies, a distinction between such cryptic species cannot be made without genetic analysis. Phylogeographic studies may help to better distinguish and identify possible cryptic species in the future. This may help to assign appropriate indicator values where populations are likely to be genetically separated. Therefore, the investigation of the cryptic species concept in relation to the indicative value of species presents a major topic for further scientific exploration. Furthermore, such future research would shed light on the relative importance and scope for investigations into other possible explanations of the observed patterns (i.e. biotic interactions and interactions between different environmental variables, as hypothesised in this study [75]).

In benthic communities, even closely related species may feed on slightly different food sources. Consequently, species are anticipated to play different role, depending on their way or in the rate at which they contribute to a distinct ecosystem feature (e.g., acting as primary producers, herbivores, predators, or detritivores) [76]. One problem is that all individuals of a given species are assumed equal, whereas characteristics such as sex and developmental stage may profoundly impact their functional roles in the ecosystem and hence the sensitivity/tolerance to stressors. However, densities and abundances are often recorded and reported with no reference to sex or developmental stage. Similarly and even more problematic, closely related species are often assumed to be equivalent (cf. expert judgement on sensitivity/tolerance), when they are clearly not [1]. The autecology of sibling species can differ completely, as they may occupy different niches. Additionally, flexible feeding strategies can vary for a species, in relation to environmental conditions [77].

Wider tolerance to salinity extremes or variability, or adaption to the lower end of the gradient is a key to surviving in low salinity waters. The horohalinicum zone (5-8 psu, sensu [78]) hosts organisms with a broad range of environmental tolerances [79] with interesting genetic implications [80]. Such waters, including inner coastal regions, are within the remit of legislative drivers such as the European Water Framework Directive. It is a paradox in estuarine quality assessment to use macroinvertebrates as biomonitors to detect pollution impacts in estuaries [3,39], since the species inventory *per se* is well adapted to this highly variable environment. Without suitable reference conditions in such highly dynamic habitats, it is often not possible to differentiate between the response due to anthropogenic disturbances and the natural fluctuation of environmental parameters.

Finally, the question arises whether simple presence/absence, species density or relative abundance is sufficient for the analysis and full understanding of environmental change. The main associated problem is that in distinct localities many of the purported indicator species naturally occur in relatively high densities [81] or have a high seasonal and spatial variability. There is no reliable methodology to know at which density level any indicator species would normally be represented in a community that is not affected by any kind of pollution [82]. Biomass or per capita biomass could give very valuable, additional information when evaluating environmental change.

Taking account of all of the above, defining ecosystem health is therefore currently mainly based on static reference lists of species (sensitivity/tolerance), because it is currently the most feasible way to summarize the ecology within a species community. This manner of ecosystem quality assessment is yet to be considered valuable and its outcomes should be seen as valid warning signals to the competent authorities. However, there is still significant scope for improvement here, which should be further explored in relation to current developments in, for example the European Water Framework Directive and Marine Strategy Framework Directive. From a scientific point of view, in relation to indicator species, attention should be paid to species autecology differentiating between developmental stages, and to the cryptic species issue in relation to indicator species, as elaborated above. This scientific progress is however only expected to gradually deliver valuable insights, which may come too late for the current implementation of environmental legislation. Pragmatically, best available expert knowledge, or evaluating the role of ‘’best professional judgment” in using indicators, may therefore be considered legitimately valuable compared to running data through statistical packages, and may be more routinely used [10,62,83,84]. We therefore suggest that in addition to significant scientific progress, the list of species sensitivities (scores, ecological groups) for marine and coastal environments be discussed among a wide group of experts during dedicated (periodic) meetings (e.g. Water Framework Directive indicator calibration meetings organised by the European Commission), rather than prepared by a few specialists. These discussions would have to focus especially on species with a wide ecological range of occurrence and tolerance, and with regard to their (functional) role in the local ecosystem and response to the environment, as illustrated in this study (Table 5). While useful, expert elicitation of best professional judgement can also fail [85]. There is unlikely to be any single approach that always works, and the incorporation of multiple lines of evidence in condition assessment is probably most powerful. A wide suite of tools, methods, and models would be best for these assessments rather than any one single indicator [37,86].

**Table 5:** Summary table of ecological requirements of selected species, given as assumptions, results and its possible explanations.

**species**	***Ampharete baltica***	***Bathyporeia pilosa***	***Cerastoderma glaucum***	***Corbula gibba***	***Hediste diversicolor***	***Travisia forbesii***
assumption	indifferent to organic load	sensitive to changes in grain size distribution	sensitive or tolerant to organic load	tolerant to organic load	tolerant to organic load	sensitive to changes in grain size distribution
result	avoidance of organic load at high salinity, higher tolerance at low salinities	large differences in preferred grain size detected between different salinity classes and ecoregions	no general sensitivity to organic load detected	found on various substrates, but absent on pure mud with high organic load	found on various substrates also with very high organic load, but highest abundances were found in substrates with medium organic load	higher sediment specificity at higher salinities, widening in mesohaline waters
rationale	different adaptation of different populations, cryptic species	apparent substrate preferences result of strong/weak interspecific competition with grain size not being the limiting parameter	different adaptation of different populations	apparent substrate preferences result of strong/weak interspecific competition	cryptic species	apparent substrate preferences result of strong/weak interspecific competition with grain size not being the limiting parameter

## Conclusion

This paper demonstrated that static indicator species lists should be applied with caution in environmental quality assessments; the importance of interacting environmental variables, the possible existence of cryptic species and the differential sensitivity of different life stages should not be neglected when using static indicator lists. In this context we do not question the use of indicator species in general, but point out several important issues that ecologist should consider before using indicator species or indices derived from them. In this work we raise the importance of when adopting such static list approach, this is applied in a context where there is a proper prior knowledge of the system and the species that are being analysed and this in cases is context (e.g. area specific). The use of benthic indicator organisms will continue to be a mainstay of marine environmental management and impact assessment. However, instead of using generic pan-European indices or species scores, the assessment schemes would benefit if dealt with in a region- and ecosystem type-specific manner. Consequently, the following points have to be considered when assessing species indicative values:

1 Large variability in patterns of species life history strategies along major natural environmental gradients and between various regions and ecosystem types;
2 Altered species autecology (and hence indicator value) along environmental gradients (e.g. salinity);
3 Need for adjustment of indicator species lists to distinct salinity/organic matter ranges;
4 Avoid pooling of sibling or cryptic species when calculating the index value of a species and/or evaluation of the uncertainty in condition assessments due to the inclusion of sibling or cryptic species in an indicator species or condition index. In most cases there is no way out of not pooling sibling or cryptic species.

We therefore recommend progressing at both the scientific level (e.g. species autecology (life-stage dependent) and the cryptic species concept (longer term perspective)) and the application level (e.g. widely solicited expert judgement on region and habitat-specific indicator values (shorter term perspective)).

## Supporting Information

Appendix S1 **Geographical coordinates of stations used for this study.**
(XLSX)

Appendix S2 **Data on sediment median grain size (d50) linked with the abundance of selected species and their salinity ranges of the Baltic Sea and North Sea.**
(XLSX)

Appendix S3 **Data on sediment organic content (LOI) linked with the abundance of selected species and their salinity ranges of the Baltic Sea and North Sea.**
(XLSX)

Appendix S4 **Data on sediment median grain size and organic content linked with the abundance of selected species and their salinity ranges of the Mediterranean Sea.**
(XLSX)
